# Portable, open-source solutions for estimating wrist position during reaching in people with stroke

**DOI:** 10.1038/s41598-021-01805-2

**Published:** 2021-11-18

**Authors:** Jeffrey Z. Nie, James W. Nie, Na-Teng Hung, R. James Cotton, Marc W. Slutzky

**Affiliations:** 1 Southern Illinois University School of Medicine, Springfield, IL 62794 USA; 2 Department of Neurology, Northwestern University, Chicago, IL 60611 USA; 3 University of Illinois at Chicago College of Medicine, Chicago, IL 60612 USA; 4 Shirley Ryan AbilityLab, Chicago, IL 60611 USA; 5 Department of Physical Medicine and Rehabilitation, Northwestern University, Chicago, IL 60611 USA; 6 Department of Neuroscience, Northwestern University, Chicago, IL 60611 USA; 7 Department of Biomedical Engineering, Northwestern University, Evanston, IL 60201 USA

**Keywords:** Biomedical engineering, Stroke

## Abstract

Arm movement kinematics may provide a more sensitive way to assess neurorehabilitation outcomes than existing metrics. However, measuring arm kinematics in people with stroke can be challenging for traditional optical tracking systems due to non-ideal environments, expense, and difficulty performing required calibration. Here, we present two open-source methods, one using inertial measurement units (IMUs) and another using virtual reality (Vive) sensors, for accurate measurements of wrist position with respect to the shoulder during reaching movements in people with stroke. We assessed the accuracy of each method during a 3D reaching task. We also demonstrated each method’s ability to track two metrics derived from kinematics-sweep area and smoothness-in people with chronic stroke. We computed correlation coefficients between the kinematics estimated by each method when appropriate. Compared to a traditional optical tracking system, both methods accurately tracked the wrist during reaching, with mean signed errors of 0.09 ± 1.81 cm and 0.48 ± 1.58 cm for the IMUs and Vive, respectively. Furthermore, both methods’ estimated kinematics were highly correlated with each other (p < 0.01). By using relatively inexpensive wearable sensors, these methods may be useful for developing kinematic metrics to evaluate stroke rehabilitation outcomes in both laboratory and clinical environments.

## Introduction

Stroke is the leading cause of long-term disability in the United States. Each year, approximately 795,000 people experience a stroke, adding to the approximately 7.5 million adults with stroke^1^. The most common deficit among people with stroke is impairment of arm or hand movement, with more than 80% of people with stroke experiencing this condition acutely and more than 40% chronically, despite standard rehabilitation^2^. Many outcome measures have been developed in an attempt to evaluate the efficacy of stroke rehabilitation methods^3^. For evaluation of motor function or impairment, several of these outcome measures, such as the Action Research Arm Test (ARAT) and Fugl-Meyer Assessment of Upper Extremity (FMA-UE), involve an observer scoring the patient's ability to perform certain tasks^4,5^. Despite widespread clinical use, most of these motor outcome measures are subject to observer bias and/or are insensitive to small changes in movement quality^6^, particularly for more severely impaired patients. Also, most functional outcome measures do not account for compensatory movements, such as movement of the trunk to reach farther, and therefore do not distinguish between compensatory movements or restitution of neural function as the explanation for functional changes^6,7^. Because these compensatory movement are common after stroke, and indeed are encouraged by some forms of physiotherapy, there is growing interest in the use of kinematic assessments as outcome measures, as they can provide objective, high-resolution quantitative measurements that account for compensatory movements^8–10^.

A number of kinematic assessments have been studied to assess upper limb motor performance in people with stroke^6,8–15^. Depending on the task, certain kinematic measures may be more informative than others. For many tasks, tracking pose (3D position and orientation) over time provides a way to directly evaluate motor function. Furthermore, changes in pose provide information from which many more kinematic metrics can be derived, such as movement speed, accuracy, smoothness, and range of motion. Determining a person’s pose involves estimating relevant joint angles and limb positions. The gold standard for pose estimation is a marker-based optical tracking system, such as the Vicon and OptiTrack motion capture systems^16^. However, these systems are expensive and require careful setup and calibration^17,18^. For the Vicon, complex static poses (e.g., T-pose, with both arms abducted and elbows straight) and dynamic range-of-motion calibration may be required depending on the skeleton model and desired task^19,20^. These movements would be difficult for many people with stroke to achieve, given frequent inability to extend the impaired elbow. Additionally, cameras have a long setup time^20^, and a large laboratory space free of vision-occluding barriers is required to accommodate the many carefully-positioned cameras. These prerequisites make the routine use of these systems to track spatial pose in people with stroke difficult, suboptimal, and infeasible for most hospital settings. Although markerless tracking systems offer the potential for less expensive setups^21,22^, these can have difficulty in measuring 3D movement, often require multiple, relatively expensive high-speed cameras, and have yet to be rigorously evaluated for accuracy. Moreover, as more rehabilitation research studies are beginning in the inpatient setting, there is a need for more portable solutions.

Inertial measurement units (IMUs) are small and easy to set up, and many approaches of varying complexity have used them to track motion in humans^23–31^. Although a full review is beyond the scope of this article, the goal of many of these approaches is to estimate joint angles and/or limb positions by utilizing the raw IMU measurements from multiple locations on the body. These raw measurements often include magnetometer, accelerometer, and gyroscope readings. However, IMUs have some disadvantages, such as susceptibility to magnetic field distortion, that can cause substantial error^32^.

Virtual reality (VR) systems provide another possible method for estimating human pose, as they inherently are designed to do this when constructing a virtual world. Here, we take advantage of this design in a consumer VR system, the HTC Vive (HTC and Valve). This system consists of small, lightweight, infrared-emitting cubes, called lighthouses, that define a trackable area (i.e., play area). The pose estimates of any Vive headset, controller, or tracker, each of which contains an array of strategically placed photodiodes, in this area are computed and tracked by the VR system. When using the trackers, the Vive was capable of estimating positions and orientations that agree with estimations made by a more expensive, marker-based optical tracking system (Vicon)^33^. Because the Vive is designed as a portable consumer device, this VR system can be used to track human pose in a variety of environments without being susceptible to magnetic fields, unlike IMUs, at the expense of a slightly longer setup time requirement.

There has been a recent call for more routine use of kinematic measures in stroke trials, as well as for more evidence of the reliability and accuracy of kinematic measurement systems^34^. However, this remains a challenge because of the paucity of validated and inexpensive methods that can practically measure kinematics at the bedside. In this study, we attempt to address this gap in the literature by developing and presenting two portable methods, one using IMUs and the other the Vive, suitable for tracking wrist position with respect to the shoulder in people with stroke. We have made our code for each method publicly available (see Code availability). Both methods first estimate the arm and forearm orientations in stroke survivors, then use these limb orientations and a kinematic model to estimate wrist position with respect to the shoulder. Although we utilized specific sensors here, our methods can be easily modified to estimate wrist position using any sensor capable of estimating limb orientation. While some commercial IMU systems provide endpoint position in Cartesian space (Xsens), they are expensive. In contrast, our IMU-based method can be used with any generic 9-axis IMU that provides magnetometer, accelerometer, and gyroscope measurements. Additionally, by tracking position relative to the shoulder, compensatory trunk movements are factored out, and a true assessment of arm mobility can be obtained. We validate each sensor-based method by comparing the magnitude of their wrist position estimations during a reaching task performed by healthy participants to estimates made using a Vicon. We derive and compare example kinematic metrics, specifically the sweep area and smoothness, from the wrist position estimations of each sensor-based method during a sweeping task performed by people with chronic stroke to demonstrate the potential clinical utility of these mobile methods.

## Results

### Validation of accuracy

**Figure 1:**
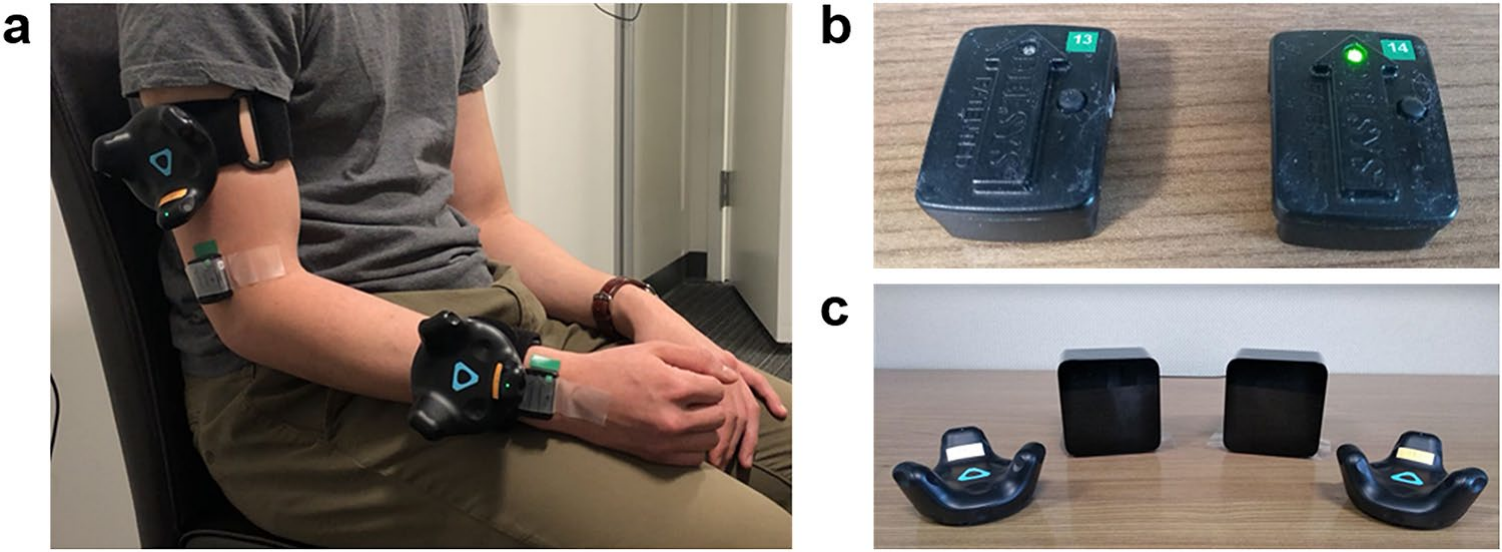
Sensors used to estimate elbow angle and wrist position. (**a**) An example IMU and Vive setup on a human participant. (**b**) The 9-axis Trigno IMUs, which also record electromyography data. (**c**) The Vive trackers (outside) and lighthouses (middle).

**Figure 2:**
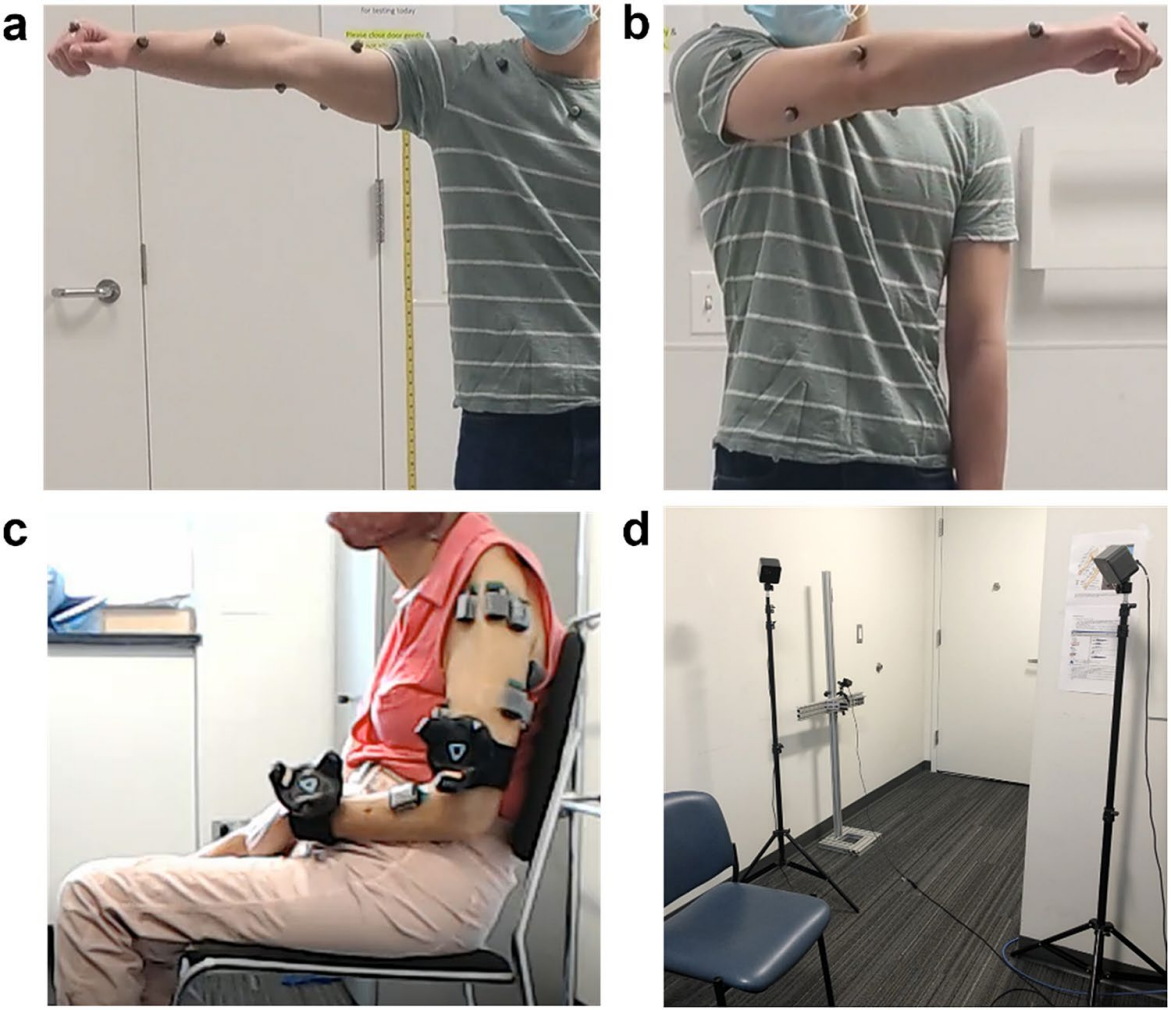
Vicon upper extremity (UE) marker setup and an example sensor setup on participants. (**a**) Vicon marker placements on the anterior arm, anterior forearm, and radial wrist. (**b**) Vicon marker placement on the posterior arm and ulnar wrist. (**c**) The IMU and Vive setup on a participant with stroke. The additional sensors on the UE are electromyography sensors. (**d**) Example Vive lighthouse positioning for tracking the left side of the participant (who would be seated in the chair as shown).

**Figure 3:**
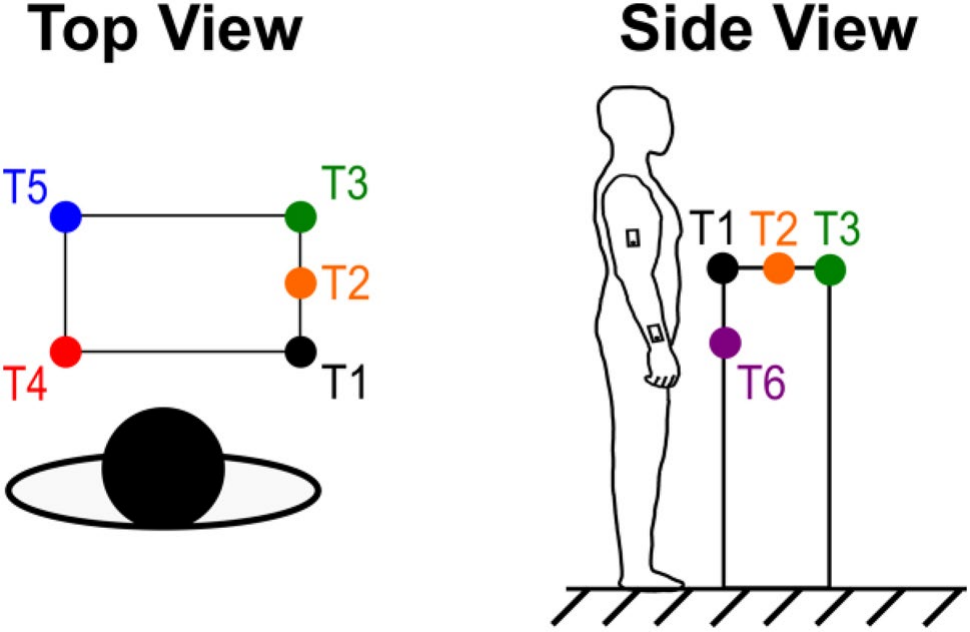
Schematic of the first task. Starting with the arm at the side, each healthy participant reached out towards target one (T1), held the reach for several seconds, then returned the arm to the side. Each participant repeated this movement sequence for the remaining targets (T2 to T6) in sequential order.

We first investigated the accuracy of the IMUs and Vive (Fig. 1) during reaching by comparing their estimations of endpoint distance (EPD), defined as the distance from the shoulder to the wrist in three dimensions, to those of a Vicon optical tracking system (Fig. 2a,b). Due to signal interference between the Vicon cameras and our first generation Vive system, it was impossible for us to record valid EPD estimations from the Vicon and Vive simultaneously. Therefore, we designed a sequence of multiplanar reaching movements that could be reliably replicated across recording sessions. Two healthy male participants performed this task on separate days. The first participant was 1.68 m tall and weighed 68.0 kg. The second participant was 1.65 m tall and weighed 61.2 kg. Each participant stood at a fixed position relative to a platform with six different marked targets within reaching distance (Fig. 3). Starting with the UE extended and at the side, each participant reached towards and touched the first target with the tip of the third finger, then maintained the reach without any radial or ulnar deviation for several seconds, and subsequently returned the UE to the initial position. This sequence was repeated 9–12 times for each target. We averaged the EPD and active range of motion (AROM), defined as the difference between the EPD prior to a reach and the EPD at the target, over all reaches per target.

We summarized these results using Bland–Altman plots. These plot the tracking error, defined as the sensor’s EPD or AROM estimation minus the Vicon’s EPD or AROM estimation for a reach, as a function of the mean EPD or AROM measurement between the two. The center line of the plot represents the mean tracking error, which indicates the degree of systemic error. A mean tracking error of 0 would suggest no systemic error and therefore complete agreement with the gold standard Vicon. The upper and lower dashed lines represent the 95% limits of agreement (mean error ± 1.96 standard deviations of the error), which indicate the degree of random (trial-to-trial) error associated with each method. A small range between the 95% limits of agreement would suggest low random error and therefore little variation in tracking error over many reaches.

**Figure 4:**
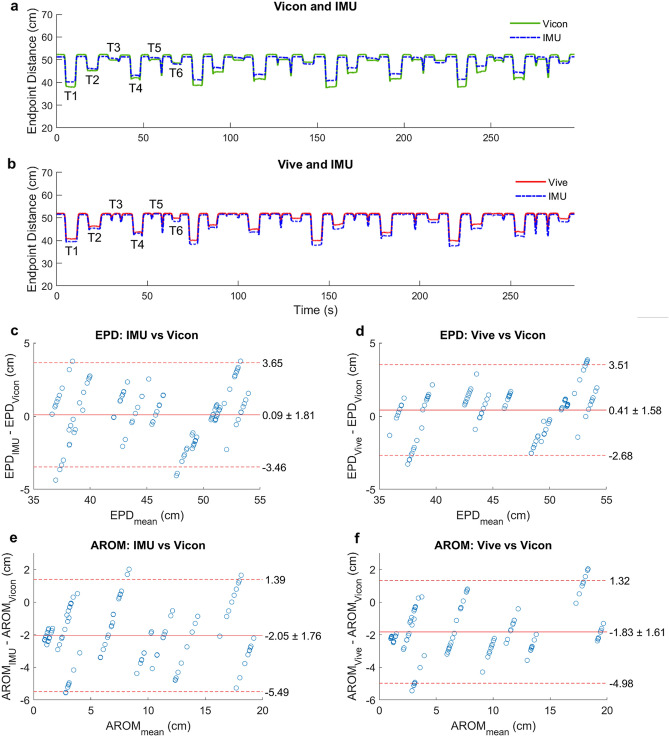
(**a**,**b**) Example of endpoint distance (EPD) tracking during the first task from four reaches to each target (24 reaches total) by healthy participant 1 while recording synchronously from the Vicon and IMU (**a**) or the Vive and IMU (**b**). The plateaus labelled T1 to T6 refer to holding at targets 1 to 6, respectively. (**c**–**f**) Bland–Altman plots comparing the IMU and Vive’s EPD and active range of motion (AROM) estimations to that of the Vicon. Each circle is from one reach.

Both IMUs and Vive tracked the EPD smoothly, without drift, and with performance similar to the Vicon throughout the reaching task (Fig. 4a,b). Both methods’ estimates of EPD and AROM remained within several cm of the Vicon estimates (Fig. 4c–f). Of note, the linear appearance of the EPD and AROM errors (Fig. 4c–f) is due to that fact that we subtracted the mean of the Vicon estimates over all reaches to a given target from each IMU and Vive estimate to that given target. Across all reaches to all targets and participants, the mean signed errors in EPD for the IMU and Vive were 0.09 ± 1.81 cm and 0.48 ± 1.58 cm, respectively. The greatest errors in EPD across all reaches and participants for the IMUs and Vive were −4.37 cm and 3.84 cm, respectively. Furthermore, the mean signed errors in AROM across all reaches to all targets for the IMU and Vive were −2.05 ± 1.76 cm and −1.83 ± 1.61 cm, respectively. The greatest errors in AROM across all reaches for the IMUs and Vive were −5.56 cm and −5.44 cm, respectively. Of note, there was variability across reaches in the Vicon estimates themselves (standard deviations ranged from 0.18 cm to 0.76 cm, see Supplementary Tables 1 and 2). Thus, some of the “error” was actually just the inherent variability among the reach distances (see “Methods” for details). That is, some of the error is just random error, rather than systemic error (seen as the spread within each cluster in Fig. 4c–f).

**Figure 5:**
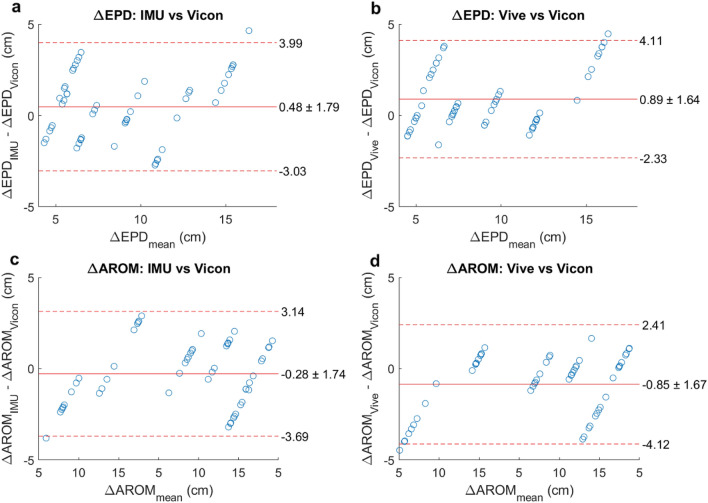
(**a**–**d**) Changes in endpoint distance (ΔEPD) and active range of motion (ΔAROM) estimated by the IMUs and Vive compared to Vicon estimates, shown as Bland–Altman plots. The changes in EPD and AROM were measured between targets one and two, targets one and three, and targets two and three.

As mentioned, we were particularly interested in how these measurements could be translated to rehabilitation outcomes, e.g., to measure improvement in reaching ability by comparing AROM to a single target pre- and post-therapy. To simulate this, we estimated the *changes* in EPD and AROM (ΔEPD and ΔAROM). We computed ΔEPD and ΔAROM by finding the difference in EPD and AROM estimates between targets one and two, targets one and three, and targets two and three. Importantly, both the IMU and the Vive were very accurate in estimating these changes (Fig. 5). The mean signed errors in ΔEPD for IMU and Vive (compared to Vicon) were 0.48 ± 1.79 cm and 0.89 ± 1.64 cm, respectively. The mean signed errors in ΔAROM for IMU and Vive were −0.28 ± 1.74 cm and −0.85 ± 1.67 cm, respectively. As in Fig. 4, the linear appearance of the ΔEPD and ΔAROM errors (Fig. 5) was due to subtracting the mean Vicon estimate from each IMU and Vive estimate.

### Estimation of example kinematic metrics

We also examined the ability of the IMU and Vive to track movement in a sweeping movement task in eight participants with arm impairment from chronic stroke (six males, two females, see “Methods”). These participants were enrolled in an ongoing clinical trial (NCT03401762) investigating a six-week training protocol of myoelectric computer interface therapy for stroke^35^. The participants’ ages ranged from 41 to 80 years old, and their FMA-UE scores ranged from 13 to 29 on the days of the task (Table 1). Four participants performed this task in one session, and four participants performed this task in two different sessions (one on weeks 4 and 6 of training, one on weeks 0 and 4, and two on weeks 0 and 6). Starting with the affected hand resting on the ipsilateral thigh while sitting, the participant attempted to abduct the affected shoulder to 90° while extending the affected elbow to 180°, then subsequently horizontally swept to the contralateral side (internally rotated the shoulder) as far as possible. This was repeated three times, yielding three “sweeps” per session. If performed by a motor-intact person, each sweep should nearly trace out a semicircle in the horizontal plane with a radius equal to the sum of the arm and forearm lengths, as the elbow should be fully extended throughout each sweep.

**Table 1:** Demographic and clinical information for participants with stroke.

	Age	Gender	Affected side	Stroke type	Months since stroke	FMA-UE W_0_	FMA-UE W_1_
Participant 1	41	M	L	Ischemic	14	–	16
Participant 2	57	F	R	Hemorrhagic	11	20	–
Participant 3	72	M	L	–	16	15	15
Participant 4	66	M	L	–	149	13	16
Participant 5	52	F	L	Hemorrhagic	94	17	–
Participant 6	80	M	L	Ischemic	56	–	–
Participant 7	46	M	R	–	69	6	–
Participant 8	50	M	L	Hemorrhagic	48	29	–

Months since stroke is the time between last known well and the baseline week W_0_. FMA-UE is the Fugl-Meyer Assessment of the upper extremity. Participants 1–4 underwent the sweeping task twice: once during the baseline week W_0_ and again during a later week W_1_. Some demographic and clinical information were not available.

The sweep task demonstrated each method’s ability to provide kinematic estimates from people with stroke that can be used to compute clinically relevant kinematic metrics. We derived two different clinically relevant metrics from the wrist position: horizontal sweep area and smoothness. This horizontal sweep area is a task-specific scalar and serves as an example kinematic metric that can be derived from the wrist position. It is effectively a 2D range of motion to all sides of the body, or a “workspace,” which makes it a functionally relevant measure^36^. We also estimated the smoothness of each sweep, a measure characteristic of unimpaired movements that has been shown to increase with stroke recovery^37,38^. We summarized these results using Bland–Altman plots.

**Figure 6:**
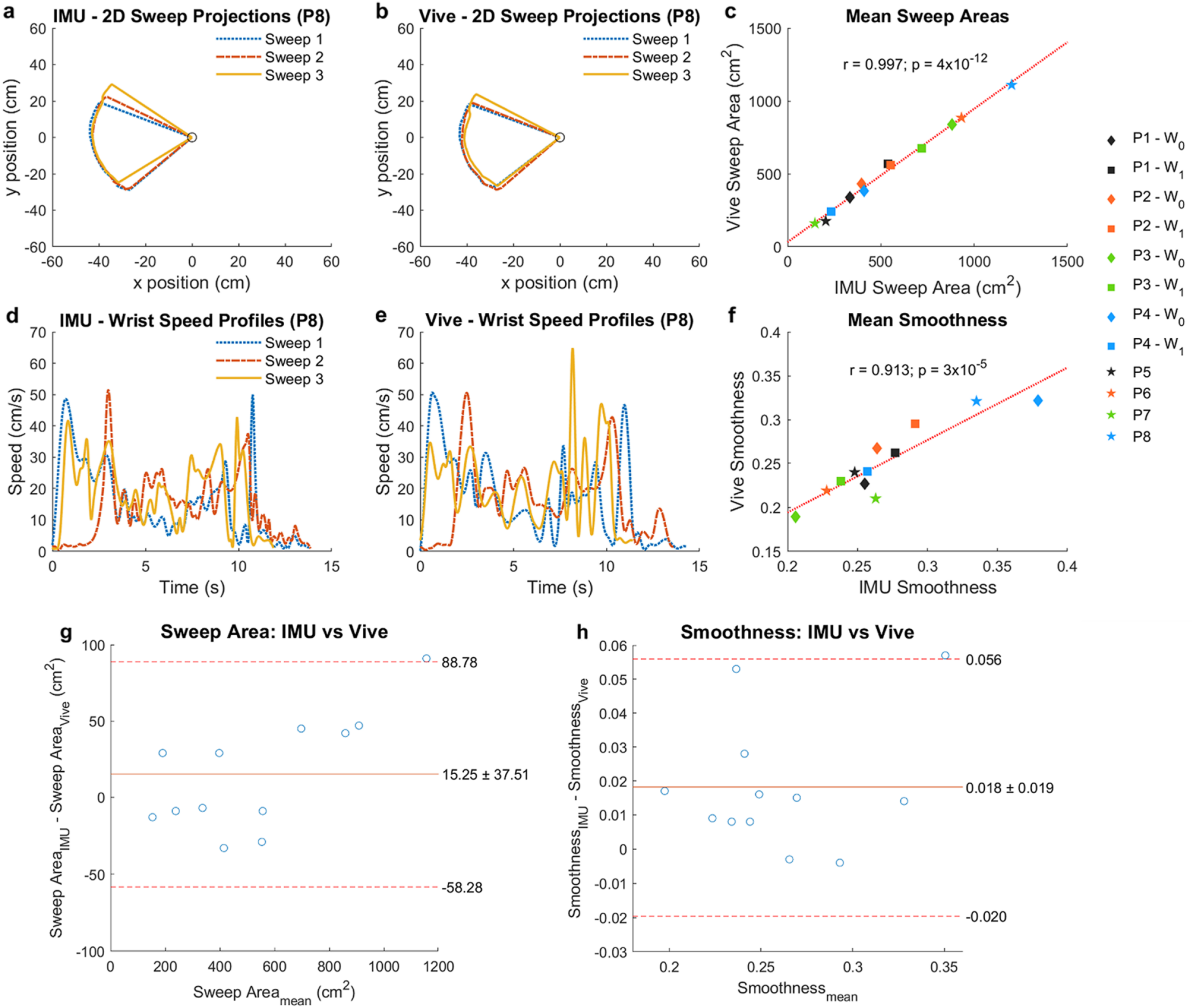
Sweep task kinematic metrics. (**a**) Example traces of the sweep areas estimated by the IMUs in participant 8 (P8). The three sweeps (blue, red, yellow) were projected onto the horizontal plane. (**b**) Example traces of the sweep areas estimated by the Vive. (**c**) Estimated mean sweep areas for all sweep tasks performed by all participants (marker labels to the right of **c** and **f**). Each marker is one mean sweep area. W_0_ and W_1_ represent the baseline week and the later week, respectively. (**d**) Example traces of wrist speed profiles estimated by the IMUs. (**e**) Example traces of the wrist speed estimated by the Vive. (**f**) Estimated smoothness for all sweep tasks. (**g**,**h**) Bland–Altman plots comparing the mean (**g**) sweep area and (**h**) smoothness estimated by the IMUs and Vive.

**Figure 7:**
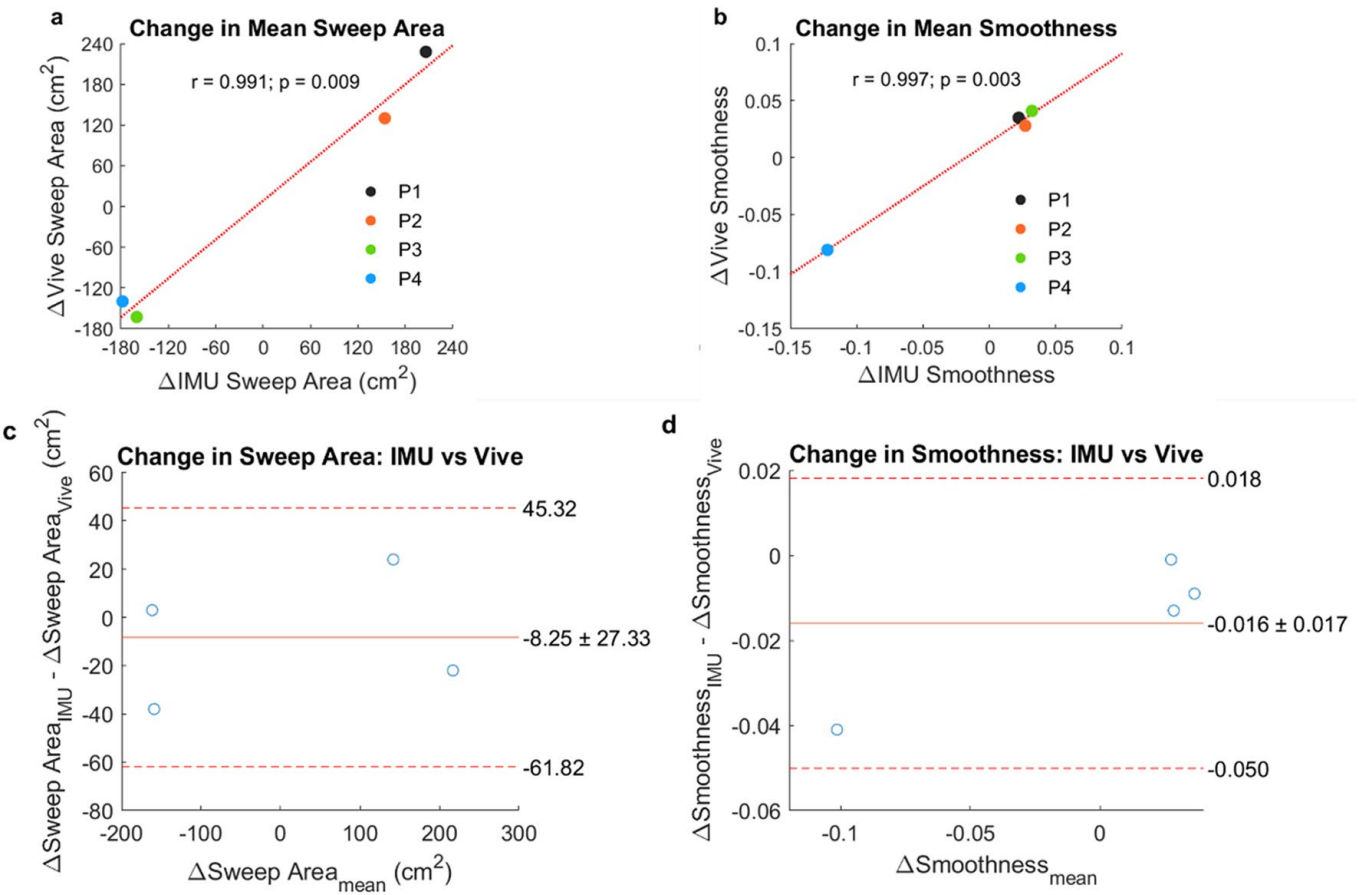
Change in sweep task kinematic metrics. (**a**,**b**) Change (Δ) in (**a**) mean sweep area and (**b**) mean smoothness between the baseline and later weeks for P1, P2, P3, and P4. **c**,**d**, Bland–Altman plots comparing the changes in (**c**) mean sweep area and (**d**) mean smoothness estimated by the IMUs and Vive.

The 2D sweep projections (Fig. 6a,b) and wrist speed profiles (Fig. 6d,e) using the IMUs and Vive agreed with each other quite well. Mean sweep areas and smoothness computed with each method were highly correlated (*r* = 0.997 and 0.913, p = 4 × 10^–12^ and 3 × 10^–5^, Fig. 6c,f) and were similar, with mean differences between the IMU and Vive of 15.25 ± 37.51 cm^2^ for sweep area and 0.018 ± 0.019 for smoothness (Fig. 6g,h). Moreover, a major goal of kinematic tracking in rehabilitation is to assess *changes* in motor performance over time. For the participants with repeated measurements, we also found that the changes in these sweep area and smoothness computed by each method between sessions were significantly correlated (*r* = 0.991 and 0.997, p = 0.009 and 0.003, respectively, Fig. 7a,b) and were similar, with mean differences between the IMU and Vive of −8.25 ± 27.33 cm^2^ for sweep area and −0.016 ± 0.017 for smoothness (Fig. 7c,d). Having sufficient sensitivity to detect changes with recovery is a key requirement for kinematic measures used in rehabilitation.

## Discussion

Here, we developed and tested two open-source methods to estimate wrist position with respect to the shoulder in people with stroke. Both the IMUs and HTC Vive can track wrist position with relatively high accuracy and precision during multiplanar UE motion. This information can be used to derive metrics of movement quality that are clinically relevant. Compared to the Vicon system, the mean EPD over all reaches and targets for the IMU and Vive were both within 1 cm, respectively. Mean AROM over all reaches and targets were within 2.5 cm of Vicon mean for both systems. Moreover, some of the error in estimation was likely just due to variability among reaches to the same target (see Vicon data in Supplementary Tables 1 and 2), as these systems were not all tested in the same session. Thus, both systems are likely even more accurate than these estimates. More importantly for clinical research, the IMU and Vive measured changes in EPD and AROM accurately, differing from Vicon estimates by less than 1 cm for both. Additionally, both methods estimated similar horizontal sweep areas and normalized mean endpoint speeds in participants with stroke and agreed on changes in these metrics over time. Thus, both the IMUs and Vive offer viable options for measuring UE kinematics in clinical research settings outside the lab. With these open-source methods, these systems can be set up in under 10 min by someone with no technical background.

Due to their portability, wearable sensor-based methods for upper extremity tracking are attractive potential alternatives to traditional optical tracking systems. One study utilized two IMUs to estimate wrist position relative to the shoulder during target reaching, reporting a root mean square error (RMSE) of 0.7 ± 1.0 cm when compared to an optical tracking system^39^. The other study presented a three-IMU method tailored for upper-extremity tracking relative to the thorax in people with impaired mobility, reporting a maximum wrist position RMSE of 1.5 ± 1.2 cm when compared to an optical tracking system during a reach-to-grasp task^40^. The wrist tracking accuracy of our IMU-based method was similar to those reported by these two studies, and we have also made our code for our IMU-based method publicly available, which can be easily adapted to work with any generic 9-axis IMU (see Code availability). In addition, we have made our Vive-based method’s code open source and, to our knowledge, were the first to investigate using the Vive as a method of estimating 3D wrist position in people with stroke. One study showed that the Vive’s accurately tracked the thorax and sacrum during reaching tasks when compared to the Vicon, reporting a mean position error within 1 cm, but the study did not evaluate tracking accuracy of the upper extremity^33^. Several studies have used VR systems as the intervention in conjunction with measuring upper extremity kinematics in stroke^12–15^, but not as the method of measuring kinematics. Similar to ours, one of these studies measured kinematics by creating a kinematic model using IMUs^13^, but they did not evaluate the accuracy of their method and used Madgwick’s gradient descent algorithm^31^ to compute IMU orientation. This algorithm, despite several merits, has several disadvantages that make it more susceptible to measurement distortion and is the predecessor to Madgwick’s improved algorithm^27^, which we used in our study. Another study utilized a kinematic model created by IMUs to assess upper extremity movement kinematics in healthy and participants with stroke performing the ARAT, but they did not validate the accuracy of their sensor fusion algorithm and kinematic model using a gold standard^41^. A third study utilized IMUs to study kinematic synergies of UE movement in healthy participants, but these sensors were from a commercial system with software dedicated to motion tracking^42^. An additional study used a IMU-based method to estimate wrist position and made their code open-source, but the system was designed for robotic applications, used a potentiometer in addition to two IMUs, utilized Madgwick’s gradient descent algorithm, required a T-pose during initialization, and did not evaluate reaching accuracy against a gold standard optical system^43^. In contrast, we evaluated the accuracy of our IMU-based method against a gold standard Vicon system during reaching.

There are several potential sources for estimation error with these systems compared to optical tracking. Both the IMUs and the Vive trackers were susceptible to misalignment with the limb, as well as to changes in alignment due to soft tissue motion relative to the underlying bone. Adapting a sensor-to-segment calibration scheme to align the sensors to the bone would help minimize this source of error. However, such schemes require the subject to perform predefined static poses or movements^44,45^, many of which would be difficult for people with stroke and moderate to severe motor impairments to perform. This can be partially alleviated by fusing video information processed with markerless pose estimation with the IMU data^46^. For the IMUs, magnetic distortion was the main source of error. Despite calibrating, the parameters were static and thus were most valid for the magnetic distortion closest to the calibration location. As the IMUs move away from the calibration location, the calibration parameters lose validity at a rate directly proportional to the degree of distortion present in the surrounding magnetic field.

We used several key methods to reduce these potential estimation error sources. These methods are described in detail in the Supplementary Information, but the following is an introduction and general overview of these key methods. First, the kinematic models for both methods only required one of the sensor’s axes to be aligned along the length of the limb. This allowed for more potential sensor attachment points around the limb’s circumference and facilitated avoiding problematic soft tissue contours (e.g., adipose tissue). Additionally, the IMU-based method incorporated baseline orientation removal and passive FE motion calibration steps into its kinematic model, which helped minimize the effects of anatomic variation and sensor alignment. Furthermore, we concatenated the resting period to the beginning of the IMU recording, and we implemented an orientation solver that estimates each sensor’s initial orientation. Both steps allowed for lower gain selection, which helped minimize the effects of distortion. Finally, the complementary filter algorithm itself helped reduce the impact of magnetic distortion by constraining the magnetometer to only influence heading (i.e. yaw)^23,27^.

Although our results show that both the IMU and Vive track wrist position with comparable accuracy, each method has important advantages and disadvantages to consider. Regarding setup and usability, the IMU’s magnetometer requires a brief calibration for accurate tracking, especially if the sensors are used in different environments. In addition, the IMU-based method requires a resting period and flexion–extension movements, which combined require approximately a minute to perform, prior to starting a task. These steps are not necessary when using the Vive. However, the Vive lighthouses require careful placement to avoid occlusion of the trackers. This may not a problem for arm motion tracking in people with stroke since the required tracking area is small; while it took us approximately a minute to properly place the lighthouses in the laboratory, this may be a consideration depending on the task and environment (e.g., may take a bit longer to set up lighthouses in a hospital room). Also, the Vive trackers are bulkier and slightly heavier than the IMUs, which may make them less suitable in people with stroke with severe arm weakness or in tighter spaces. Both the IMUs and Vive may be suitable for hospital rooms, but the Vive may be better if the environment is less controllable in terms of metal objects. Finally, the IMU used in this study can record electromyography data synchronously, which could be advantageous for some studies.

As described in the “Introduction”, the Vicon system requires extensive setup and calibration before use. Prior to data collection, we typically spent 30 to 60 min to place the markers, perform an initial calibration procedure to confirm camera functionality, and verify that the cameras detect the markers. However, we found that small, occasionally trivial disturbances in the setup, such as slamming a door or accidently bumping a reflective marker, adversely affected Vicon estimations and required a shorter 5-to-10-min recalibration procedure before resuming data collection. These disadvantages, as well as the difficulty of transporting the hardware itself, makes routine use of the Vicon in different locations challenging. Compared to the Vicon and other optical tracking systems, relatively minimal alignment and calibration steps are required for the IMUs and Vive, typically taking at most 10 min, and the calibration motions were achievable by people with stroke. This made motion tracking less burdensome on the patient and more convenient for the researcher or clinician. Furthermore, both systems are compact, lightweight, and relatively easy to set up. This permits UE motion tracking for patients outside of the laboratory environment, such as in an inpatient setting early after stroke. Finally, traditional optical tracking systems and commercial systems with software that performs sensor fusion and motion tracking (e.g. Xsens) typically cost at least tens of thousands of dollars. The IMUs used in this study and the Vive are considerably less expensive, and our IMU-based method can be used with any generic 9-axis IMU, making them more accessible to researchers and clinicians, especially in underdeveloped countries and underserved communities.

Multiple commercial markerless video motion capture systems, perhaps the most often studied being the Microsoft Kinect, can also provide motion tracking for biomechanical and clinical applications^47–49^. The Kinect position tracking errors vary with distance from the camera, but one study in motor-intact participants showed mean errors for single upper extremity joint distances within 2–3 cm^50^, which was stated to be well within rehabilitation needs^51^. This is similar to the error range of both of our methods, although we cannot make a direct comparison due to the differences in our task, distance metrics, and gold standard selection. However, another study compared the Kinect to the Vicon for motion tracking in healthy individuals and individuals with Parkinson’s disease, finding that the Kinect performed relatively poorly during upper extremity movements^52^. These systems often rely on light in the visual and infrared spectra for motion tracking^48,49^, which makes them susceptible to both changes in lighting conditions and occlusion. Additionally, pose estimated from 2D color images are first expressed in terms of pixels, which needs to be converted to a real-world distance to be clinically relevant (including measuring exact distances to the patient, and ensuring that the patient’s positioning is exactly the same from session to session). Moreover, the Microsoft Kinect has been replaced by the Azure Kinect, which needs to be warmed up for at least 40 min to provide stable results^53^, and is no longer commercially available. These requirements may make it difficult to use these systems to track movement practically in a hospital environment. An emerging method for markerless vision-based pose estimation is the use of deep neural networks to estimate pose from 2D images^21,22,54,55^, which could be promising for clinical environments. One study utilized this method in a hospital setting, but it only measured 2D pose and required manually labeled training data in each patient to account for environmental variations and to ensure proper tracking of a broad range of postures^22^. Further, only one study has tracked 3D pose using a stereoscopic camera system in a laboratory environment, but accuracy was not rigorously evaluated^21^. These studies did not directly evaluate accuracy in terms of absolute Cartesian distance (e.g., in cm) or compare to a gold standard optical tracking system, making the accuracy results less clinically relevant. Finally, these techniques are also subject to potential occlusion, depending on the type of motion made. Thus, it remains to be seen how markerless motion tracking will compare to attachable external sensors in terms of performance, as well as cost.

This study has several limitations. First, despite carefully designing and executing the reaching task, the Vive was compared to the Vicon asynchronously due to hardware incompatibilities, precluding a rigorous comparison of accuracy when performing tasks at different speeds. Also, these methods were only tested in a laboratory environment. Furthermore, testing was done on a small number of subjects, and all participants with stroke had severe upper extremity impairment. Moreover, it is worth highlighting that there is additional kinematic information estimated that we did not analyze in this study, including elbow angle and position. Future work should include evaluating these methods in a hospital environment, incorporating a sensor-to-segment calibration scheme compatible with people with stroke and motor impairments, recording data from people with stroke with a wider range of functional ability, and studying additional clinically relevant kinematic metrics using these methods.

Kinematic outcome metrics are an important, emerging technique for assessing stroke rehabilitation that may provide more sensitive and specific outcome measures than commonly used clinical scores. Importantly, these methods can remove confounds from compensatory movements that may distort functional scores. Although not the end goal of this study, we showed two examples of functionally relevant kinematic metrics that can be derived from the 3D wrist position vector estimated by these methods, the sweep area (i.e. reaching workspace)^36^ and smoothness^37^. These methods can be extended to estimate numerous other kinematic metrics relevant to stroke survivors used in prior studies^8^. For example, the wrist position vector can be differentiated to obtain any velocity-based metric, such as peak velocity and mean velocity. Additionally, the wrist position vector can be plotted over time to determine trajectory-based metrics, such as trajectory error and reach-path-ratio. It remains to be seen what types of kinematic metrics will be most useful for identifying improvement after stroke rehabilitation.

## Methods

All procedures were approved by the Northwestern University Institutional Review Board and performed in accordance with the Declaration of Helsinki. Informed consent was obtained from all participants in the study. To evaluate the accuracy of each tracking method, we recorded data using 9-axis IMUs (Trigno IM Sensor, Delsys Inc., Fig. 1b), Vive (Fig. 1c), and Vicon (Fig. 2a,b) from two healthy participants. To demonstrate the utility of the IMUs and Vive, we also recorded data from eight participants with stroke using these sensors in the laboratory during reaching tasks (detailed in System evaluation).

### Estimating wrist position

We provide a general overview of the common aspects of both the IMU- and Vive-based methods in the following section. More details and recording procedures are described in the Supplementary Information. Prior to taking any sensor measurements, we performed the following critical steps to help ensure proper estimation of wrist position: measuring the length of the arm and forearm and aligning the sensors on the limbs. We measured the arm from the acromion to the antecubital fossa, and we measured the forearm from the antecubital fossa to the center of the ventral aspect of the wrist. We placed the arm and forearm sensors proximal to the elbow and wrist, respectively, and aligned one axis of each sensor with the long axis of the respective limb (Fig. 1a). This step ensured that the directions of the arm and forearm were always known, regardless of each limb’s orientation in space.

We then determined the 3D orientations of the arm and forearm sensors to estimate each limb’s 3D orientation. For the IMUs, we implemented a variant of the complementary filter to estimate sensor orientation from the raw measurements. For the Vive, we simply needed to position the lighthouses such that the trackers were not occluded from the lighthouses (Fig. 2d), as the Vive automatically estimated sensor orientation. We then used these quantities, as well as the measured limb lengths, to construct a serial kinematic chain model that models the upper extremity (UE) and estimates the wrist position with respect to the shoulder, denoted as the position vector ${{\mathbf{p}}_{\mathbf{w}\mathbf{r}\mathbf{i}}}^{\mathbf{w}}$. Note that the wrist position is a function of the arm and forearm orientations, which can be determined using any type of sensor-based method. We obtained recordings from the IMUs and Vive using the recording procedures described in the IMU-based Tracking and Vive-based Tracking sections of the Supplementary Information.

### Accuracy testing

The Vicon system was set up in an enclosed room with eight Vicon Vantage cameras surrounding the workspace. We calibrated the cameras using the system’s specified procedure^20^. We placed reflective markers corresponding to the Vicon UE model onto two healthy human participants (Fig. 2a,b)^19^ on different days. Each participant performed a calibration procedure (static pose with shoulder abducted and elbow extended, Fig. 2a) optimized for the Vicon system prior to performing the desired task^19,20^. All Vicon data were sampled at 200 Hz and imported into MATLAB for analysis.

We collected data from the first healthy participant across four different recording sessions on one day: one with Vicon (four reaches to target 1, five reaches to remaining targets), one with both IMU and Vicon (five reaches to all targets), one with both IMU and Vive (four reaches to all targets), and one with Vive (five reaches to all targets). The combined Vicon data contained nine reaches to target 1 and 10 reaches to each remaining target (59 total reaches). We collected data from the second healthy participant across two different recording sessions on another day: one with Vicon (12 reaches to each target, total of 72 reaches) and one with both IMU and Vive (nine reaches to each target, total of 54 reaches). Throughout each recording session, we computed the EPD—i.e., the scalar distance from the shoulder to the wrist—by taking the magnitude of the estimated position vector ${{\mathbf{p}}_{\mathbf{w}\mathbf{r}\mathbf{i}}}^{\mathbf{w}}$. By doing so, we were able to compare position estimations between the sensors, as the orientations of the reference frames in which ${{\mathbf{p}}_{\mathbf{w}\mathbf{r}\mathbf{i}}}^{\mathbf{w}}$ was expressed in were different for each sensor. We averaged all samples while maintaining a reach at a target to estimate the EPD at that target for a given reach. We averaged the Vicon-estimated EPDs over reaches to each target to obtain the ground truth mean EPD and AROM estimations for each target. Next, we determined the IMU and Vive’s estimated EPD at each target for all 54 (nine per target) reaches and subtracted them from the corresponding ground truth mean EPD. Likewise, we computed the IMU and Vive’s estimated AROM for each reach and subtracted them from the corresponding ground truth AROM. We then computed the mean and standard deviation of the these differences in estimated EPD and AROM. Because we subtracted each IMU/Vive estimate from the mean of the Vicon estimates to each target, this metric was subject to some jitter due to variability across reaches, as mentioned in the “Discussion”; this was unavoidable due to the inability to simultaneously record Vicon and Vive. Finally, we computed the differences in the estimated mean AROM between the reaches to targets one, two, and three for all three methods. These differences allowed us to measure and compare how sensitive each method was at detecting *changes* in AROM in a given direction. These changes simulated how the methods could detect improved AROM from a course of therapy; in the case of people with stroke, this would mean reaching to a single target in two different sessions and computing the change in AROM. We summarized these results using Bland–Altman plots with 95% limits of agreement^56^.

### Estimation of example kinematic metrics

For the sweep task, we placed IMUs and Vive trackers on the affected limbs of eight people with chronic stroke with UE motor impairment (Fig. 2c). We computed the horizontal sweep area by first projecting ${{\mathbf{p}}_{\mathbf{w}\mathbf{r}\mathbf{i}}}^{\mathbf{w}}$ onto the horizontal plane and then using MATLAB’s *polyarea* function. After differentiating all ${{\mathbf{p}}_{\mathbf{w}\mathbf{r}\mathbf{i}}}^{\mathbf{w}}$ estimations from the initiation (relaxed state immediately prior to shoulder abduction) to termination (relaxed state immediately after maximal shoulder internal rotation) of the sweep using smooth noise-robust differentiators^57^, we computed the magnitude of resulting 3D velocity vector to obtain the endpoint speed (EPS). Afterwards, we extracted the peak envelope of the EPS using MATLAB’s *envelope* function (spline interpolation over local maxima) to reduce noise introduced by filter gain selection and numerical differentiation while maintaining the overall shape of the speed profile. We applied the same peak envelope to both the IMU and Vive data. From the peak envelope of the EPS, we computed the normalized mean endpoint speed (i.e., smoothness), defined as the max EPS divided by the mean EPS during the sweep^37^.

After estimating both kinematic metrics for each sweep, we calculated and compared the mean of each metric over all sweeps. We also computed Pearson’s correlation coefficient (using MATLAB’s *corrcoef* function) between the IMU and Vive’s estimated mean for each metric and defined significance as p < 0.05. For the four chronic stroke participants who performed the task in two different sessions, we also computed the change over time in the estimated metrics for each method and compared them. We summarized these results using Bland–Altman plots.

### Informed consent

All participants were admitted to the study following informed consent as approved by the Northwestern University Institutional Review Board.

## Supplementary Information

Supplementary Information.

## Data Availability

The code used to estimate wrist position using the two methods are available at https://github.com/JeffNie96/IMU-Vive-Kinematics. The datasets used and/or analyzed during the current study are available from the corresponding author on reasonable request.
