# Supplementary material for: Portable, open-source solutions for estimating wrist position during reaching in people with stroke
Source: Sci Rep. 2021 Nov 18;11:22491. doi: 10.1038/s41598-021-01805-2 (PMC8602299; doi:10.1038/s41598-021-01805-2)
Supplement: Supplementary file 1 — Supplementary Information. [file 41598_2021_1805_MOESM1_ESM.docx]

**Portable, open-source solutions for estimating wrist position during reaching in people with stroke – Supplementary Information**

Jeffrey Z. Nie*^1,2^, James W. Nie^2,3^, Na-Teng Hung^2,4^, R. James Cotton^4,5^, Marc W. Slutzky*^2,4,5,6,7^

^1^ Southern Illinois University School of Medicine, Springfield, IL 62794, United States of America

^2^ Department of Neurology, Northwestern University, Chicago, IL 60611, United States of America

^3^ University of Illinois at Chicago College of Medicine, Chicago, IL 60612, United States of America

^4^ Shirley Ryan AbilityLab, Chicago, IL 60611, United States of America

^5^ Department of Physical Medicine and Rehabilitation, Northwestern University, Chicago, IL 60611, United States of America

^6^ Department of Neuroscience, Northwestern University, Chicago, IL 60611, United States of America

^7^ Department of Biomedical Engineering, Northwestern University, Evanston, IL 60201, United States of America

* Please direct all correspondence concerning this manuscript to Jeffrey Z. Nie at [jnie31@siumed.edu](mailto:jnie31@siumed.edu) and Marc W. Slutzky at [mslutzky](mailto:jnie31@siumed.edu)@northwestern.edu

**METHODS**

**Common Background**

Regardless of type, each sensor has a coordinate frame rigidly attached to the sensor, denoted as frame *A* and frame *FA* for the arm and forearm sensors, respectively, that defines its 3D orientation. There are numerous ways to parameterize 3D orientations, such as the roll-pitch-yaw Euler angles, and many of these parameterizations, their operations and conversions, and their advantages and disadvantages can be found in reference^1^. We utilized several different parameterizations, starting with the quaternion. The quaternion is defined as the following unit length 4D vector

$$\begin{aligned} \mathbf{q}_{\mathbf{b}}^{\mathbf{a}}=\left[ q_{0},q_{1},q_{2},q_{3} \right]^{T}=\left[ \cos\left( \frac{\alpha}{2} \right),n_{x}\sin\left( \frac{\alpha}{2} \right),n_{y}\sin\left( \frac{\alpha}{2} \right),n_{z}\sin\left( \frac{\alpha}{2} \right) \right]^{T} \boldsymbol{\#}\left( 1 \right) \end{aligned}$$

where$\mathbf{q}_{\mathbf{b}}^{\mathbf{a}}$ describes the rotation from frame *a* to frame *b*, $\mathbf{n}\boldsymbol{=}\left[ n_{x},n{}_{y},n_{z} \right]^{T}$ is the axis of rotation as a unit vector, and $\alpha$ is the scalar amount of rotation about $\mathbf{n}$. Since we placed a sensor on the arm and forearm, we estimated the orientation of each limb by computing the quaternions $\mathbf{q}_{\mathbf{A}}^{\mathbf{w}}$ and $\mathbf{q}_{\mathbf{FA}}^{\mathbf{w}}$, which describe the arm and forearm sensor frames with respect to the world frame *w* (origin at the shoulder), respectively. We then computed the relative orientation between the arm and forearm sensors, $\mathbf{q}_{\mathbf{FA}}^{\mathbf{A}}$, using

$$\begin{aligned} \mathbf{q}_{\mathbf{FA}}^{\mathbf{A}}=\mathbf{q}_{\mathbf{w}}^{\mathbf{A}}\circ\mathbf{q}_{\mathbf{FA}}^{\mathbf{w}}\mathbf{=}\left( \mathbf{q}_{\mathbf{A}}^{\mathbf{w}} \right)^{\mathbf{*}}\circ\mathbf{q}_{\mathbf{FA}}^{\mathbf{w}}\boldsymbol{\#}\left( 2 \right) \end{aligned}$$

where $\circ$ represents quaternion multiplication, and * represents the quaternion conjugate.

We then used these quantities, as well as the measured limb lengths, to construct a series of 4x4 homogenous transformation matrices that modeled the UE as a serial kinematic chain model. We multiplied these transformation matrices to compute the net transformation matrix, $\mathbf{T}_{\mathbf{wri}}^{\mathbf{w}}$, which contains the position vector from the shoulder to the wrist expressed in the world frame, denoted as ${\mathbf{p}_{\mathbf{wri}}}^{\mathbf{w}}$. Obtaining $\mathbf{q}_{\mathbf{A}}^{\mathbf{w}}$ and $\mathbf{q}_{\mathbf{FA}}^{\mathbf{w}}$ and creating the kinematic model differed between methods and are discussed below.

**Vive-Based Tracking**

In this study, we used first generation lighthouses and trackers. Each Vive lighthouse sweeps alternating horizontal and vertical IR lasers that are detected by the photodiode-containing trackers within the play area. The time delay from the onset of each sweep emitted from the fixed lighthouses to detection by the photodiodes allows determination of the position and orientation of any tracker in the play area. For evaluating arm kinematics, the required play area is relatively small (1.5x1.5 m) since participants can perform arm movements while standing or sitting in a fixed location.

We positioned the lighthouses such that the trackers were always visible to them. A common acceptable setup was having two lighthouses approximately two meters apart face each other on an oblique plane relative to the patient (Figure 2d). This was critical to obtain valid pose estimations from the Vive, as failure to do so would increase the chance of occlusion (i.e., lighthouse could not detect the trackers) and therefore introduce potentially large, unpredictable error in pose estimation. Since the required play area was relatively small, it was easy to find an appropriate lighthouse setup. Once placed on the limb, each tracker had an associated position, ${\mathbf{r}_{\mathbf{FA}}}^{\mathbf{w}}$ and ${\mathbf{r}_{\mathbf{A}}}^{\mathbf{w}}$, and orientation, $\mathbf{q}_{\mathbf{FA}}^{\mathbf{w}}$ and $\mathbf{q}_{\mathbf{A}}^{\mathbf{w}}$, estimation automatically expressed with respect to a common world frame. We neglected the position estimations because they can sometimes become unstable during movement^2^. Using the SteamVR Unity Plugin (found in the Unity assets store), we loaded virtual representations of the Vive trackers into Unity 3D to observe their position and orientation in real time. We then extracted and recorded the orientation estimations of each tracker, as well as time stamps synchronized with the computer’s clock because the sampling rate varied with the frame rate of Unity, into text files using the Unity Scripting Application Programming Interface. We subsequently imported these files into MATLAB and interpolated the data to a sampling rate of 50 Hz.


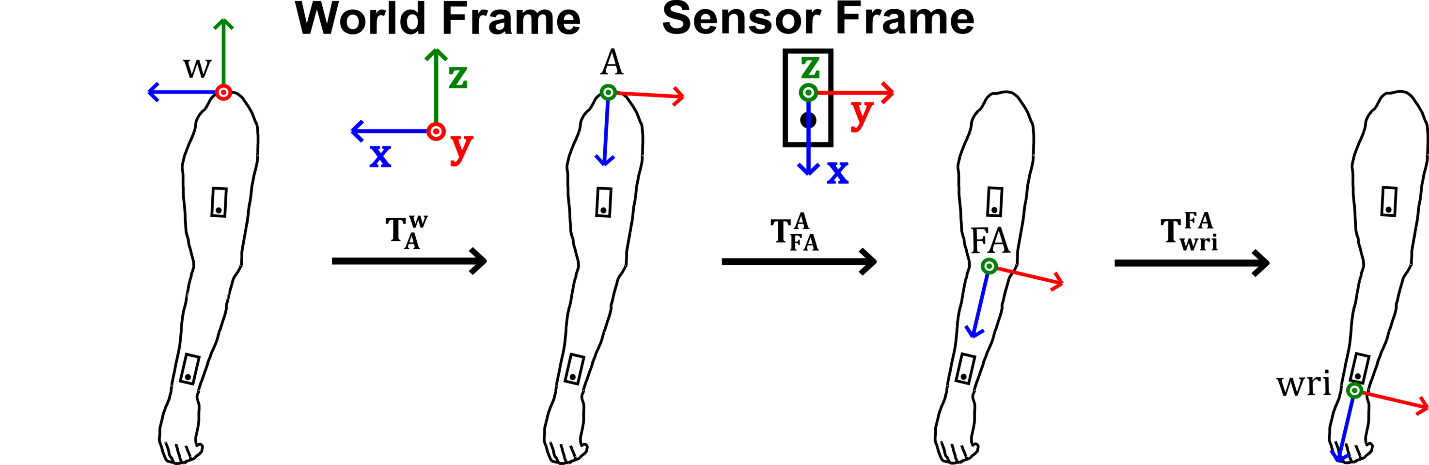


**Supplemental Figure S1.** Sequence of three transformations for the Vive that transforms a world frame (*w*, left) with origin at the shoulder to a frame aligned with the forearm with origin at the wrist (*wri*, right). *A*, true arm frame (aligned with the arm sensor); *FA*, true forearm frame (aligned with the forearm sensor); T, transformation matrices.

We constructed a kinematic chain model using 4x4 homogenous transformation matrices and solved the forward kinematics problem^3^ to estimate the wrist position with respect to the shoulder, ${\mathbf{p}_{\mathbf{wri}}}^{\mathbf{w}}$ (Supplementary Fig. S1). This sequence transforms the world frame with origin at the acromion to a frame partially aligned with the forearm sensor and with origin at the wrist. This final frame has only partial alignment with the forearm sensor because our model does not incorporate the degree of PS. This is because physically pronating or supinating the forearm generally does not change the actual wrist position in space, so the PS angle is not necessary to compute ${\mathbf{p}_{\mathbf{wri}}}^{\mathbf{w}}$ and therefore does not need to be incorporated into the kinematic model.

The first transformation matrix in this sequence, $\mathbf{T}_{\mathbf{A}}^{\mathbf{w}}$, is

$$\begin{aligned} \mathbf{T}_{\mathbf{A}}^{\mathbf{w}}\boldsymbol{=}\left[ \begin{matrix} \mathbf{R}_{\mathbf{A}}^{\mathbf{w}} & \boldsymbol{0}_{\boldsymbol{3}\mathbf{x1}} \\ \boldsymbol{0}_{\boldsymbol{1}\mathbf{x3}} & 1 \end{matrix} \right] \boldsymbol{\#}\left( 3 \right) \end{aligned}$$

where $\mathbf{R}_{\mathbf{A}}^{\mathbf{w}}$ is the equivalent rotation matrix representation of $\mathbf{q}_{\mathbf{A}}^{\mathbf{w}}$. Assuming that the x-axis of the arm sensor is aligned with the length of the arm, the second transformation matrix, $\mathbf{T}_{\mathbf{FA}}^{\mathbf{A}}$, is

$$\begin{aligned} \mathbf{T}_{\mathbf{FA}}^{\mathbf{A}}\boldsymbol{=}\left[ \begin{matrix} \mathbf{R}_{\mathbf{FA}}^{\mathbf{A}} & {\mathbf{p}_{\mathbf{A}}}^{\mathbf{A}} \\ \boldsymbol{0}_{\mathbf{1x3}} & 1 \end{matrix} \right] \boldsymbol{\#}\left( 4 \right) \end{aligned}$$

where $\mathbf{R}_{\mathbf{FA}}^{\mathbf{A}}$ is the rotation matrix representation of $\mathbf{q}_{\mathbf{FA}}^{\mathbf{A}}$, ${\mathbf{p}_{\mathbf{A}}}^{\mathbf{A}}\boldsymbol{=}\left[ l_{A}\boldsymbol{,}0, 0 \right]^{T}$ is the vector containing the translation from the acromion to the antecubital fossa, and $l_{A}$ is the measured arm length. Assuming that the x-axis of the forearm sensor is aligned with the length of the forearm, the final transformation, $\mathbf{T}_{\mathbf{wri}}^{\mathbf{FA}}$, is

$$\begin{aligned} \mathbf{T}_{\mathbf{wri}}^{\mathbf{FA}}\boldsymbol{=}\left[ \begin{matrix} \mathbf{I}_{\mathbf{3x3}} & {\mathbf{p}_{\mathbf{FA}}}^{\mathbf{FA}} \\ \boldsymbol{0}_{\mathbf{1x3}} & 1 \end{matrix} \right] \boldsymbol{\#}\left( 5 \right) \end{aligned}$$

where $\mathbf{I}_{\mathbf{3x3}}$ is the 3x3 identity matrix, ${\mathbf{p}_{\mathbf{FA}}}^{\mathbf{FA}}\boldsymbol{=[}l_{FA}\boldsymbol{,}0,0\boldsymbol{]}$ is the vector containing the translation from the antecubital fossa to the center of the wrist, and $l_{FA}$ is the measured forearm length. We multiplied these three transformation matrices to obtain $\mathbf{T}_{\mathbf{wri}}^{\mathbf{w}}$.

A typical recording session using the Vive proceeded as follows. We first positioned two lighthouses to capture a sufficiently large play area. We confirmed this by moving the trackers in the play area and observing the real-time movement of their virtual representations in Unity without recording data. If the virtual representations ceased to move simultaneously with tracker movement, signifying tracker occlusion, we adjusted the position of the lighthouses until they redetected the trackers. Afterwards, we performed the limb measurement and sensor alignment steps as described in *Common Background*. Because there was no required data calibration step, the participant simply proceeded with the assessment once the data recording began. Overall, the Vive setup and calibration steps usually took at most 5 minutes to perform.

**IMU-Based Tracking**

For the IMU-based method, we used a 9-axis IMU containing a 3-axis accelerometer, 3-axis magnetometer, and 3-axis gyroscope (Trigno IM Sensor, Delsys Inc.) to record raw inertial data. We imported the raw IMU data into MATLAB and resampled the accelerometer and gyroscope data to the magnetometer’s sampling frequency of 74 Hz for analysis.

The accelerometer and magnetometer are both susceptible to measurement distortions that produce error in orientation estimation. For the accelerometer, non-gravitational accelerations (e.g., ballistic movements) partially mask gravity. In motor-impaired stroke survivors, this distortion is usually small due to generally low-acceleration movements. For the magnetometer, metal alloys in nearby objects, such as in walls, floors, beds, and tables, can distort the magnetic field^4^. Because these distortions can have magnitudes of a substantial fraction of the Earth’s magnetic field, the magnetometer is much more susceptible to measurement distortion than the accelerometer. Therefore, it is critical to calibrate the magnetometer whenever the local magnetic environment changes. We calibrated the magnetometer by recording while rotating the IMUs by 90° increments four times about the sensor’s positive and negative *x*, *y*, and *z* axes, which typically took approximately three minutes. We performed this calibration before putting the IMUs on the arm and forearm. We then used a least-squares ellipsoid fitting method to find the parameters that transformed the recorded magnetometer measurements into a sphere^5,6^. Afterwards, we used the recorded accelerometer recordings to calibrate the accelerometer and remove any cross-axis misalignment between the accelerometer and magnetometer sensor axes^7^.

Following calibration, we fused the accelerometer, gyroscope, and magnetometer measurements to estimate sensor orientation $\mathbf{q}_{\mathbf{s}}^{\mathbf{w}}$ by implementing a modified version of the improved explicit complementary filter^8,9^. Complementary filters (CFs) are widely used to compute orientation using 9-axis IMU measurements^8–11^ because they select only the low frequency, stable components of the accelerometer/magnetometer estimations and the high frequency, drift-free components of the gyroscope estimations. We used this specific version due to several important features, including no singularities, flexible gain selection, gyroscope bias compensation, and decoupled magnetometer influence from roll and pitch estimations^8,9^.

Let $\boldsymbol{\omega}^{\mathbf{s}}=\left[ \omega_{x}, \omega_{y},\omega_{z} \right]^{T}$be the gyroscopic angular velocity expressed in a sensor frame, either the arm or the forearm. The quaternion rate is computed by the following expression:

$$\begin{aligned} \dot{\mathbf{q}_{\mathbf{s}}^{\mathbf{w}}}=\frac{1}{2}\mathbf{q}_{\mathbf{s}}^{\mathbf{w}}\boldsymbol{\circ}\left[ \begin{matrix} 0 \\ \boldsymbol{\omega}^{\mathbf{s}} \end{matrix} \right] \boldsymbol{\#}\left( 6 \right) \end{aligned}$$

This quantity can then be numerically integrated to yield $\mathbf{q}_{\mathbf{s}}^{\mathbf{w}}$ using

$\begin{aligned} {\mathbf{q}_{\mathbf{s}}^{\mathbf{w}}}_{k+1}\mathbf{=}{\mathbf{q}_{\mathbf{s}}^{\mathbf{w}}}_{k}+{\dot{\mathbf{q}_{\mathbf{s}}^{\mathbf{w}}}}_{k}T_{s} \boldsymbol{\#}\left( 7 \right) \end{aligned}$

where $T_{s}$ is the sampling period. This computation yields an accurate and smooth short-term estimation of $\mathbf{q}_{\mathbf{s}}^{\mathbf{w}}$. However, this estimation deviates considerably from the true value of $\mathbf{q}_{\mathbf{s}}^{\mathbf{w}}$after a short period of time due to integration drift, making it unsuitable for elbow angle tracking beyond a few seconds. Solutions to Wahba’s problem^12^ show that vector observations of at least two reference vectors are required to fully determine the 3D orientation of body^13,14^. The accelerometer measurements $\mathbf{a}^{\mathbf{s}}=\left[ a_{x}, a_{y},a_{z} \right]^{T}$of the gravity vector and the magnetometer measurements $\mathbf{m}^{\mathbf{s}}=\left[ m_{x}, m_{y},m_{z} \right]^{T}$of the Earth’s magnetic field can serve as these vector observations. Since gravity and the magnetic field vectors remain constant in the North-East-Down world frame, the orientation can be determined without use of integration. This yields a stable, drift-free estimation of $\mathbf{q}_{\mathbf{s}}^{\mathbf{w}}$. However, this estimation is noisy and becomes inaccurate as soon as measurement distortions are present, such as large non-gravity accelerations or nearby magnetic disturbances.

Fusing the accelerometer, gyroscope, and magnetometer measurements is therefore critical to obtain an accurate and stable estimate of $\mathbf{q}_{\mathbf{s}}^{\mathbf{w}}$. To do so, we implemented the Improved Explicit Complementary Filter described in^8^ with slight modifications from^9^. Our implementation of this filter can be summarized by the following equations:

$$\begin{aligned} {\mathbf{q}_{\mathbf{s}}^{\mathbf{w}}}_{k+1}\mathbf{=}{\mathbf{q}_{\mathbf{s}}^{\mathbf{w}}}_{k}+{\dot{\mathbf{q}_{\mathbf{s}}^{\mathbf{w}}}}_{CF, k}T_{s} \boldsymbol{\#}\left( 8 \right) \end{aligned}$$

$$\begin{aligned} {\dot{\mathbf{q}_{\mathbf{s}}^{\mathbf{w}}}}_{\mathrm{CF}}\boldsymbol{=}\frac{1}{2}\mathbf{q}_{\mathbf{s}}^{\mathbf{w}}\boldsymbol{\circ}\left[ \begin{matrix} 0 \\ \boldsymbol{\omega}^{\mathbf{s}}-\boldsymbol{\omega}_{\mathbf{bias}}+K_{aq}\mathbf{e}_{\mathbf{a}}+K_{mq}\mathbf{e}_{\mathbf{m}} \end{matrix} \right] \boldsymbol{\#}\left( 9 \right) \end{aligned}$$

$$\begin{aligned} \boldsymbol{\omega}_{\mathbf{bias,}k+1}=\boldsymbol{\omega}_{\mathbf{bias,}k}+K_{a\omega}\mathbf{e}_{\mathbf{a}}+K_{m\omega}\mathbf{e}_{\mathbf{m}}\boldsymbol{\#}\left( 10 \right) \end{aligned}$$

$$\begin{aligned} \mathbf{e}_{\mathbf{a}}=\frac{\mathbf{a}^{\mathbf{s}}}{\left\| \mathbf{a}^{\mathbf{s}} \right\|}{\times\mathbf{R}}_{\mathbf{w}}^{\mathbf{s}}\mathbf{g}\boldsymbol{\#}\left( 11 \right) \end{aligned}$$

$$\begin{aligned} \mathbf{e}_{\mathbf{m}}=\frac{\mathbf{a}^{\mathbf{s}}\times\mathbf{m}^{\mathbf{s}}}{\left\| \mathbf{a}^{\mathbf{s}}\times\mathbf{m}^{\mathbf{s}} \right\|}{\times\mathbf{R}}_{\mathbf{w}}^{\mathbf{s}}\frac{\mathbf{g}\times\mathbf{m}^{\mathbf{w}}}{\left\| \mathbf{g}\times\mathbf{m}^{\mathbf{w}} \right\|} \boldsymbol{\#}\left( 12 \right) \end{aligned}$$

Here,${\dot{\mathbf{q}_{\mathbf{s}}^{\mathbf{w}}}}_{CF, k}$ is the corrected quaternion rate at sample k; $\boldsymbol{\omega}_{\mathbf{bias}}$ is the gyroscope bias estimation; $K_{aq}$, $K_{mq}$,$K_{a\omega}$, and $K_{m\omega}$are gains; $\mathbf{e}_{\mathbf{a}}$ and $\mathbf{e}_{\mathbf{m}}$ are the accelerometer and magnetometer errors, respectively; $\mathbf{R}_{\mathbf{w}}^{\mathbf{s}}$ is the rotation matrix representation of $\mathbf{q}_{\mathbf{w}}^{\mathbf{s}}$ (i.e. the quaternion conjugate of $\mathbf{q}_{\mathbf{s}}^{\mathbf{w}}$); $\mathbf{g}$ is the gravity vector in the world frame (i.e. $\left[ 0, 0, g \right]^{T}$); and $\mathbf{m}^{\mathbf{w}}$ is the Earth’s magnetic field expressed in the world frame, determined using the World Magnetic Model^15^. Conceptually, this uses the accelerometer and magnetometer measurements to estimate the IMU’s inclination and heading, respectively. Scaled by a gain, these two measurements constitute a “ground truth” 3D orientation that reduces drift from directly integrating the gyroscope. The gain selection is critical. Higher gains increase the influence of the accelerometer and magnetometer and thus the “ground truth” 3D orientation, resulting in greater suppression of drift but making the final orientation estimation $\mathbf{q}_{\mathbf{s}}^{\mathbf{w}}$ noisier and more susceptible to measurement distortions, such as large non-gravitational accelerations or nearby magnetic disturbances.

We generally set the gains to be as low as possible after considering the calibration and raw measurement quality to maximize the gyroscope’s influence on the estimation of $\mathbf{q}_{\mathbf{s}}^{\mathbf{w}}$ without observing drift, instability (e.g. oscillations), or nonconvergence, as the gyroscope is much less sensitive to distortions than the accelerometer and magnetometer^9^. In general, lower filter gains weigh the gyroscope estimations more, reducing noise and the influence of accelerometer and magnetometer distortions but increasing drift. Additionally, we fed the initial magnetometer and accelerometer measurements into the Optimal Quaternion Estimation algorithm^14^ to obtain a close initial quaternion for faster convergence. This allowed for lower gain selection, which helped minimize effects of accelerometer and magnetic distortion when necessary.

When we initially placed the sensors on the limbs, there was a static baseline orientation difference between the two sensors. We removed this baseline orientation to ensure that only FE and PS about the elbow joint contributed to differences in the relative orientation between the two sensors^9^. Before performing any motion, the initial estimations of the relative orientation, $\mathbf{(q}_{\mathbf{FA}}^{\mathbf{A}}\mathbf{)}{}_{\mathbf{initial}}$, equaled the baseline orientation difference. Thus, the quaternion from the world frame to the arm sensor frame after baseline orientation removal, $\mathbf{q}_{\mathbf{Ai}}^{\mathbf{w}}$, is defined as

$$\begin{aligned} \mathbf{q}_{\mathbf{Ai}}^{\mathbf{w}}=\mathbf{q}_{\mathbf{A}}^{\mathbf{w}}\circ\mathbf{(q}_{\mathbf{FA}}^{\mathbf{A}}\mathbf{)}{}_{\mathbf{initial}}\boldsymbol{\#}\left( 13 \right) \end{aligned}$$

where frame *Ai* represents a virtual, intermediate arm frame that initially has the same orientation as frame *FA* during baseline orientation removal. Note that this operation removes any initial orientation difference between the two sensors. With equation (10), we computed subsequent relative orientations $\mathbf{q}_{\mathbf{FA}}^{\mathbf{Ai}}$, representing only motion about the elbow joint, by modifying equation (2) to the following:

$$\begin{aligned} \mathbf{q}_{\mathbf{FA}}^{\mathbf{Ai}}=\mathbf{q}_{\mathbf{w}}^{\mathbf{Ai}}\circ\mathbf{q}_{\mathbf{FA}}^{\mathbf{w}}\mathbf{=}\left( \mathbf{q}_{\mathbf{Ai}}^{\mathbf{w}} \right)^{\mathbf{*}}\circ\mathbf{q}_{\mathbf{FA}}^{\mathbf{w}} \boldsymbol{\#}\left( 14 \right) \end{aligned}$$

After obtaining $\mathbf{q}_{\mathbf{FA}}^{\mathbf{Ai}}$, the next step was to determine the FE axis of rotation $\mathbf{j}$ in the sensor frames. If calibration motion to determine $\mathbf{j}^{\mathbf{FA}}$ was performed at the same PS angle as when baseline orientation was removed, then $\mathbf{j=}\mathbf{j}^{\mathbf{Ai}}\mathbf{=}\mathbf{j}^{\mathbf{FA}}$. We determined $\mathbf{j}$ using the method described in ^16^. This method utilizes the fact that the direction of $\boldsymbol{\omega}^{\mathbf{s}}$ is parallel to **j** during pure FE motion of the elbow. Thus, the following statements are always true during pure FE motion for any IMU sensor:

$$\begin{aligned} \left\| \boldsymbol{\omega}^{\mathbf{s}}\times\mathbf{j} \right\|=0 \#\left( 15 \right) \end{aligned}$$

$$\begin{aligned} \frac{d\left\| \boldsymbol{\omega}^{\mathbf{s}}\times\mathbf{j} \right\|}{d\mathbf{j}}=\frac{\left( \boldsymbol{\omega}^{\mathbf{s}}\boldsymbol{\times}\mathbf{j} \right)\times\boldsymbol{\omega}^{\mathbf{s}}}{\left\| \boldsymbol{\omega}^{\mathbf{s}}\times\mathbf{j} \right\|}=0 \#\left( 16 \right) \end{aligned}$$

Given a data set of pure FE motion, $\mathbf{j}$ can be solved for by minimizing equation (12) using the Gauss-Newton algorithm. Once we obtained $\mathbf{j}$, we converted $\mathbf{q}_{\mathbf{FA}}^{\mathbf{Ai}}$ to the equivalent $\mathbf{v}_{\mathbf{FA}}^{\mathbf{Ai}}$, where $\mathbf{v}_{\mathbf{FA}}^{\mathbf{Ai}}$ is the 3D rotation vector parameterization of the relative orientation^1^. With $\mathbf{v}_{\mathbf{FA}}^{\mathbf{Ai}}$ and $\mathbf{j}$, the rotation along the FE axis to get from frame *Ai* to frame *FA* is

$$\begin{aligned} d\theta=\mathbf{v}_{\mathbf{FA}}^{\mathbf{Ai}}\cdot\mathbf{j} \#\left( 17 \right) \end{aligned}$$

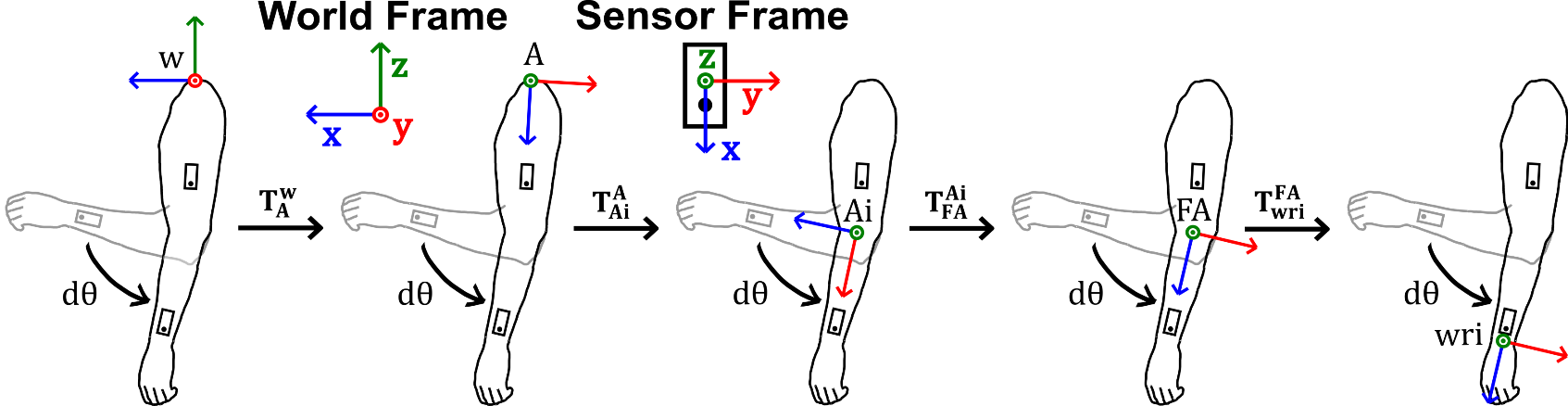


**Supplemental Figure S2.** Sequence of four transformations for the IMUs that transforms a world frame (*w*, left) with origin at the shoulder to a frame aligned with the forearm with origin at the wrist (*wri*, right). The gray limbs represent the limb orientations when the baseline orientation difference was removed (i.e. initial orientation), and the darker limbs represent an extension of the elbow by $d\theta$ from the initial state. *A*, true arm frame (aligned with the arm sensor); *Ai*, intermediate arm frame (aligned with the forearm sensor initially); *FA*, true forearm frame (aligned with the forearm sensor); T, transformation matrices.

We constructed a kinematic chain model using 4x4 homogenous transformation matrices and solved the forward kinematics problem^3^ to estimate the wrist position with respect to the shoulder, ${\mathbf{p}_{\mathbf{wri}}}^{\mathbf{w}}$ (Supplementary Fig. S2). This sequence transforms the world frame with origin at the acromion to a frame partially aligned with the forearm sensor and with origin at the wrist. This final frame has only partial alignment with the forearm sensor because our model does not incorporate the degree of PS. This is because physically pronating or supinating the forearm generally does not change the actual wrist position in space, so the PS angle is not necessary to compute ${\mathbf{p}_{\mathbf{wri}}}^{\mathbf{w}}$ and therefore does not need to be incorporated into the kinematic model.

The first transformation matrix in this sequence, $\mathbf{T}_{\mathbf{A}}^{\mathbf{w}}$, is defined by equation (3). The next transformation matrix, $\mathbf{T}_{\mathbf{Ai}}^{\mathbf{A}}$, is

$$\begin{aligned} \mathbf{T}_{\mathbf{Ai}}^{\mathbf{A}}=\left[ \begin{matrix} \mathbf{(R}_{\mathbf{FA}}^{\mathbf{A}}\mathbf{)}{}_{\mathbf{initial}} & {\mathbf{p}_{\mathbf{A}}}^{\mathbf{A}} \\ \boldsymbol{0}_{\boldsymbol{1}\mathbf{x3}} & 1 \end{matrix} \right] \boldsymbol{\#}\left( 18 \right) \end{aligned}$$

where $\mathbf{(R}_{\mathbf{FA}}^{\mathbf{A}}\mathbf{)}{}_{\mathbf{initial}}$ is the rotation matrix representation of $\mathbf{(q}_{\mathbf{FA}}^{\mathbf{A}}\mathbf{)}{}_{\mathbf{initial}}$. Note that this rotation makes ${\mathbf{p}_{\mathbf{wri}}}^{\mathbf{w}}$ a function of $d\theta$. Assuming that the x-axis of the arm sensor is aligned with the length of the arm, ${\mathbf{p}_{\mathbf{A}}}^{\mathbf{A}}\boldsymbol{=}\left[ l_{A}\boldsymbol{,}0, 0 \right]^{T}$ is the vector containing the translation from the acromion to the antecubital fossa, and $l_{A}$ is the measured arm length. The following transformation matrix, $\mathbf{T}_{\mathbf{FA}}^{\mathbf{Ai}}$, is defined as

$$\begin{aligned} \mathbf{T}_{\mathbf{FA}}^{\mathbf{Ai}}\boldsymbol{=}\left[ \begin{matrix} \mathbf{R}_{\mathbf{FA}}^{\mathbf{Ai}} & \boldsymbol{0}_{\boldsymbol{3}\mathbf{x}\boldsymbol{1}} \\ \boldsymbol{0}_{\mathbf{1x3}} & 1 \end{matrix} \right] \boldsymbol{\#}\left( 19 \right) \end{aligned}$$

where $\mathbf{R}_{\mathbf{FA}}^{\mathbf{Ai}}$ is the rotation matrix describing a rotation of $d\theta$ about the FE axis of rotation $\mathbf{j}$. Assuming that the x-axis of the forearm sensor is aligned with the length of the forearm, the final transformation, $\mathbf{T}_{\mathbf{wri}}^{\mathbf{FA}}$, is defined by equation (5). Multiplying these four transformation matrices, the net transformation matrix, $\mathbf{T}_{\mathbf{wri}}^{\mathbf{w}}$, is

$$\begin{aligned} \mathbf{T}_{\mathbf{wri}}^{\mathbf{w}}\boldsymbol{=}\mathbf{T}_{\mathbf{A}}^{\mathbf{w}}\mathbf{T}_{\mathbf{Ai}}^{\mathbf{A}}\mathbf{T}_{\mathbf{FA}}^{\mathbf{Ai}}\mathbf{T}_{\mathbf{wri}}^{\mathbf{FA}}\boldsymbol{=}\left[ \begin{matrix} \mathbf{R}_{\mathbf{wri}}^{\mathbf{w}} & {\mathbf{p}_{\mathbf{wri}}}^{\mathbf{w}} \\ \boldsymbol{0}_{\mathbf{1x3}} & 1 \end{matrix} \right] \boldsymbol{\#}\left( 20 \right) \end{aligned}$$

where ${\mathbf{p}_{\mathbf{wri}}}^{\mathbf{w}}$ is the position vector from the shoulder to the wrist expressed in the world frame.

Regarding the data calibration procedure for a typical recording session, maintaining the static, neutral pose for 30-60 seconds provided raw IMU measurements that we used while processing the data. Specifically, we concatenated these raw IMU measurements to the beginning of the recorded data to extend the period available for filter convergence. This allowed us to select lower gains in the setting of more severe magnetometer and accelerometer distortion. Additionally, this static period provided $\mathbf{q}_{\mathbf{A}}^{\mathbf{w}}$ and $\mathbf{q}_{\mathbf{FA}}^{\mathbf{w}}$ estimations for baseline orientation removal. Furthermore, the subsequent passive FE motion provided gyroscope data for determining $\mathbf{j}$.

A typical recording session using the IMU proceeded as follows. Prior to recording participant data, we calibrated the sensors in the testing environment. Afterwards, we performed the limb measurement and sensor alignment steps as described in *Common Background*. At the start of data collection, the participant performed a brief calibration procedure necessary for developing the kinematic chain model (i.e., data calibration procedure). This consisted of a static, neutral pose for 30-60 seconds orientation estimations for baseline orientation removal, followed by passive flexion-extension (FE) four times without pronation-supination (PS) motion. After the data calibration procedure, the participant relaxed and then performed the assessment tasks. Overall, the IMU setup and calibration steps usually took on average approximately 10 minutes to perform.

**RESULTS**

**Accuracy Testing**

For each healthy participant, we computed the mean endpoint distance (EPD) and active range of motion (AROM) estimated by the Vicon over reaches to each target and defined these values as each target’s ground truth EPD and AROM. We defined EPD as the magnitude of the position vector between the shoulder and wrist. We defined AROM as the difference between the EPD at rest prior to reaching and the EPD upon reaching a target. We then computed the mean EPD and AROM error per target, defined as the mean difference between the IMUs or Vive’s EPD and AROM estimates and the ground truth EPD and AROM (Supplemental Tables 1 and 2).

**Supplemental Table 1.** Estimation accuracy of the IMU and Vive for the first healthy participant.

| Target | Vicon EPD (cm) | Vicon AROM (cm) | EPD error (cm) | | AROM error (cm) | |
| --- | --- | --- | --- | --- | --- | --- |
|  |  |  | IMU | Vive | IMU | Vive |
| Target 1 | 38.61 ± 0.28 | 14.77 ± 0.26 | 1.22 ± 1.40 | 1.24 ± 0.45 | -3.23 ± 1.48 | -2.76 ± 0.41 |
| Target 2 | 45.74 ± 0.54 | 7.54 ± 0.61 | 0.37 ± 0.72 | 1.24 ± 0.37 | -2.30 ± 0.77 | -2.67 ± 0.39 |
| Target 3 | 50.80 ± 0.23 | 2.47 ± 0.25 | 0.26 ± 0.36 | 0.79 ± 0.30 | -2.14 ± 0.31 | -2.23 ± 0.20 |
| Target 4 | 42.13 ± 0.47 | 11.21 ± 0.47 | 1.16 ± 0.75 | 1.47 ± 0.61 | -3.19 ± 0.79 | -2.95 ± 0.58 |
| Target 5 | 51.18 ± 0.18 | 2.13 ± 0.28 | 0.14 ± 0.32 | 0.75 ± 0.05 | -2.03 ± 0.25 | -2.17 ± 0.05 |
| Target 6 | 50.13 ± 0.52 | 3.31 ± 0.58 | -1.84 ± 0.22 | -0.95 ± 0.47 | -0.29 ± 0.30 | -0.56 ± 0.45 |
| Vicon endpoint distance (EPD) and active range of motion (AROM) are the means and standard deviations of the Vicon’s estimations across all nine reaches per target. EPD/AROM errors are the mean and standard deviation of the differences between the mean Vicon EPD/AROM and the IMU and Vive EPD/AROM estimations for all reaches per target. | | | | | | |

**Supplemental Table 2.** Estimation accuracy of the IMU and Vive for the second healthy participant.

| Target | Vicon EPD (cm) | Vicon AROM (cm) | EPD error (cm) | | AROM error (cm) | |
| --- | --- | --- | --- | --- | --- | --- |
|  |  |  | IMU | Vive | IMU | Vive |
| Target 1 | 39.14 ± 0.71 | 17.31 ± 0.76 | -2.21 ± 1.43 | -2.37 ± 0.72 | 0.20 ± 1.14 | 1.26 ± 0.68 |
| Target 2 | 43.90 ± 0.42 | 12.40 ± 0.60 | 0.18 ± 1.39 | 0.36 ± 0.68 | -2.23 ± 1.08 | -1.51 ± 0.61 |
| Target 3 | 53.19 ± 0.38 | 3.57 ± 0.42 | 0.18 ± 1.07 | 0.75 ± 0.96 | -1.71 ± 0.99 | -1.83 ± 0.95 |
| Target 4 | 36.57 ± 0.58 | 20.33 ± 0.74 | 1.55 ± 1.13 | 0.25 ± 0.68 | -3.40 ± 0.93 | -1.78 ± 0.58 |
| Target 5 | 51.41 ± 0.47 | 5.59 ± 0.67 | 2.73 ± 0.82 | 3.23 ± 0.71 | -4.86 ± 0.73 | -4.81 ± 0.67 |
| Target 6 | 49.65 ± 0.64 | 7.34 ± 0.60 | -2.59 ± 0.96 | -1.80 ± 0.65 | 0.48 ± 0.88 | 0.07 ± 0.63 |
| Vicon endpoint distance (EPD) and active range of motion (AROM) are the means and standard deviations of the Vicon’s estimations across all nine reaches per target. EPD/AROM errors are the mean and standard deviation of the differences between the mean Vicon EPD/AROM and the IMU and Vive EPD/AROM estimations for all reaches per target. | | | | | | |

**References**

1. Diebel, J. Representing Attitude: Euler Angles, Unit Quaternions, and Rotation Vectors. (2006). doi:10.1093/jxb/erm298

2. Borges, M., Symington, A., Coltin, B., Smith, T. & Ventura, R. HTC Vive: Analysis and Accuracy Improvement. in *2018 IEEE/RSJ International Conference On Intelligent Robots and Systems (IROS)* 2610–2615 (2018).

3. Waldron, K. & Schmiedeler, J. Kinematics. in *Springer Handbook of Robotics* (eds. Siciliano, B. & Khatib, O.) 9–33 (Springer, Berlin, Heidelberg, 2016). doi:10.5555/3002858

4. de Vries, W. H. K., Veeger, H. E. J., Baten, C. T. M. & van der Helm, F. C. T. Magnetic distortion in motion labs, implications for validating inertial magnetic sensors. *Gait Posture* **29**, 535–541 (2009).

5. Li, Q. & Griffiths, J. G. Least Squares Ellipsoid Specific Fitting. in *Geometric Modeling and Processing, 2004, Proceedings* 335–340 (2004). doi:10.1109/gmap.2004.1290055

6. Afzal, M. H. Complete Triaxis Magnetometer Calibration in the Magnetic Domain. *J. Sensors* **2010**, (2010).

7. Tomczynski, J., Mankowsi, T. & Kaczmarek, P. Cross-Sensor Calibration Procedure for Magnetometer and Inertial Units. in *Advances in Intelligent Systems and Computing* (eds. Szewczyk, R., Zieliński, C. & Kaliczyńska, M.) **550**, 450–459 (Springer, Cham, 2017).

8. Fan, B., Li, Q. & Liu, T. How Magnetic Disturbance Influences the Attitude and Heading in Magnetic and Inertial Sensor-Based Orientation Estimation. *Sensors* **18**, (2018).

9. Cotton, R. J. & Rogers, J. Wearable Monitoring of Joint Angle and Muscle Activity. in *Proceedings of the 16th IEEE International Conference on Rehabilitation Robotics (ICORR)* 258–263 (2019).

10. Valenti, R. G., Dryanovski, I. & Xiao, J. Keeping a Good Attitude: A Quaternion-Based Orientation Filter for IMUs and MARGs. *Sensors* **15**, 19302–19330 (2015).

11. Wu, J., Zhou, Z., Chen, J., Fourati, H. & Li, R. Fast Complementary Filter for Attitude Estimation Using Low-Cost MARG Sensors. *IEEE Sens. J.* **16**, 6997–7007 (2016).

12. Wahba, G. Problem 65-1 : A Least Squares Estimate of Satellite Attitude. *SIAM Rev.* **7**, 409 (2007).

13. Shuster, M. D. & Oh, S. D. Three-Axis Attitude Determination from Vector Observations. *J. Guid. Control. Dyn.* **4**, 70–77 (1981).

14. Markley, F. L. Fast Quaternion Attitude Estimation from Two Vector Measurements. *J. Guid. Control. Dyn.* **25**, 411–414 (2002).

15. National Oceanic and Atmospheric Administration. World Magnetic Model. Available at: https://www.ngdc.noaa.gov/geomag/calculators/magcalc.shtml#igrfwmm.

16. Seel, T., Schauer, T. & Raisch, J. Joint Axis and Position Estimation from Inertial Measurement Data by Exploiting Kinematic Constraints. in *Proceedings from the 2012 IEEE International Conference on Control Applications* 45–49 (2012). doi:10.1109/CCA.2012.6402423
